# Rates of protein synthesis are maintained in brain but reduced in skeletal muscle during dietary sulfur amino acid restriction

**DOI:** 10.3389/fragi.2022.975129

**Published:** 2022-08-24

**Authors:** Wenceslao Martinez, Qian Zhang, Melissa A. Linden, Nate Schacher, Sanna Darvish, Emily T. Mirek, Jordan L. Levy, William O. Jonsson, Tracy G. Anthony, Karyn L. Hamilton

**Affiliations:** ^1^ Department of Health and Exercise Science, Colorado State University, Fort Collins, CO, United States; ^2^ Department of Nutritional Sciences and the New Jersey Institute for Food, Nutrition and Health, Rutgers University, New Brunswick, NB, United States; ^3^ Columbine Health Systems Center for Healthy Aging, Colorado State University, Fort Collins, CO, United States

**Keywords:** methionine restriction, amino acids, healthspan, proteostasis, energy sensing, dietary intervention

## Abstract

Dietary interventions such as sulfur amino acid restriction (SAAR) target multiple drivers of aging, and show promise for preventing or delaying the onset of chronic diseases. SAAR promotes metabolic health and longevity in laboratory animals. The effects of SAAR on proteostasis remain relatively unexplored. We previously reported that SAAR promotes mitochondrial proteostatic maintenance, despite suppression of global protein synthesis, in two peripheral tissues, the liver and skeletal muscle. However, the brain, a tissue vulnerable to age-related neurodegenerative diseases due to the loss of proteostasis, has not been thoroughly studied. Therefore, we sought to reveal proteostatic responses in the brains of mice fed SAAR for 35 days. Here, we demonstrate that male C57Bl/6J mice fed two levels of SAAR maintained rates of protein synthesis in all sub-cellular fractions of the pre-frontal cortex. In comparison, rates of skeletal muscle protein synthesis in SAAR fed mice were slower than control-fed mice. To gain mechanistic insight, we examined several key nutrient/energy sensitive signaling proteins: AMP-activated protein kinase (AMPK), eukaryotic initiation factor 2 (eIF2), and ribosomal protein S6 (rpS6). SAAR had minimal to modest effects on the total abundance and phosphorylation of these proteins in both tissues. Our results indicate that the pre-frontal cortex in brain is resistant to perturbations in protein synthesis in mice fed SAAR, unlike skeletal muscle, which had a reduction in global protein synthesis. The results from this study demonstrate that proteostatic control in brain is of higher priority than skeletal muscle during dietary SAAR.

## Introduction

Advancing age is the predominant risk factor for many chronic diseases including cardiovascular diseases, cancer, and neurodegenerative diseases (Niccoli and Partridge, 2012). Lifestyle factors including dietary choices can contribute to disease development. Additionally, healthspan-extending dietary interventions can be powerful strategies to delay or prevent the onset of chronic diseases by targeting multiple interconnected drivers of cellular aging (Lopez-Otin et al., 2013; Li et al., 2021).

One dietary intervention called Sulfur Amino Acid Restriction (SAAR), extends longevity and improves metabolic health in laboratory models (Orentreich et al., 1993; Miller et al., 2005; Lee et al., 2014). SAAR involves eliminating cysteine and lowering the amount of methionine ∼80% [from 0.86 to ∼0.17 g per 100 g experimental diet (Jonsson et al., 2019)]. In addition to extending lifespan, dietary SAAR reduces bodyweight and adiposity, and increases energy expenditure (Anthony et al., 2013). While the metabolic effects of SAAR are dependent on the level of SAA restriction, they are not counteracted by increases in dietary lipid but instead alleviate high fat diet-induced cognitive and metabolic dysfunctions (Forney et al., 2017; Yang et al., 2019; Wang et al., 2020a; Wang et al., 2020b). The mechanisms by which SAAR may extend healthspan have been studied in peripheral tissues including liver and muscle. For example, in older mice on a high fat diet, SAAR maintains or improves thyroid function, oxidative defenses, glucose tolerance, and muscle mass (Yang et al., 2018; Swaminathan et al., 2021). The liver, is especially positioned and primed to sense and evoke a response to SAAR (Stone et al., 2021). Detection of amino acid insufficiency in the liver occurs via activation of general control nonderepressible 2 (GCN2) kinase, which phosphorylates eukaryotic initiation factor 2 (eIF2) on its alpha subunit (Zhang et al., 2002). eIF2 phosphorylation directs preferential translation of activating transcription factor 4 (ATF4), which orchestrates cellular responses involved in amino acid and lipid metabolism, and antioxidant defenses (Vattem and Wek, 2004). However, the metabolic effects of SAAR can occur independently of GCN2 and eIF2, prompting investigation of other mediators (Wanders et al., 2016; Pettit et al., 2017; Jonsson et al., 2021). Within hours of initiating dietary SAAR, the liver releases fibroblast growth factor 21 (FGF21), an ATF4 target gene that is postulated to mediate some of the metabolic and cognitive effects of SAAR (Forney et al., 2020; Ren et al., 2021).

Protein homeostasis (proteostasis), is the dynamic regulation of protein synthesis, folding, trafficking, and degradation. Loss of proteostasis is a hallmark of aging and of many chronic diseases (Santra et al., 2019). Because mechanisms of protein repair are limited, protein turnover is necessary to degrade and replace damaged proteins to maintain proteostasis (Chondrogianni et al., 2014). Protein turnover is influenced by energetic/nutrient status. Cells undergoing nutrient stresses including amino acid insufficiency, typically have increased phosphorylation of eIF2, which suppresses global protein synthesis in order to maintain the amino acid pool (Baird and Wek, 2012). We previously showed that mice fed SAAR diets demonstrate lower rates of hepatic mixed and cytosolic protein synthesis as compared to mice fed a control diet; however, hepatic mitochondrial protein synthesis rates were maintained (Pettit et al., 2017). A reduction in protein synthesis could be attributed to repression of an important regulator of translation, the mechanistic target of rapamycin complex 1 (mTORC1), through GCN2 or ATF4 activation (Ye et al., 2015; Nikonorova et al., 2018; Jonsson et al., 2022). However, the role of AMPK and downstream effectors of mTORC1 such as ribosomal protein S6 (rpS6) have not been extensively studied with SAAR.

The influence of SAAR on rates of protein synthesis has been previously studied in energetically active tissues including liver and muscle (Pettit et al., 2017; Jonsson et al., 2021). Only one study has measured the impact of dietary SAAR on protein synthesis in the brain, reporting no differences in hippocampal protein synthesis after 16 days of SAAR (Nichenametla et al., 2018). It is not known how SAAR impacts rates of protein synthesis, cellular proliferation, and key energy sensing proteins in the pre-frontal cortex of brain, a region of the brain important for cognition that undergoes a decline in function and volume during aging (Nyberg et al., 2010). Given that the loss of proteostasis is culprit in neurodegenerative diseases, and SAAR has been shown to improve cognitive function, it is important to understand proteostatic responds to SAAR in the brain (Kurtishi et al., 2019; Wang et al., 2020a; Ren et al., 2021). The purpose of this brief research study is to assess the impact of dietary SAAR on rates of protein synthesis and cellular proliferation measured simultaneously in the pre-frontal cortex, and on activation of key nutrient sensing proteins involved in regulating protein synthesis.

## Materials and methods

### Animals

Male wild-type C57BL/6J mice (Jackson Laboratory) aged 3–6 months were housed at Rutgers University in conventional shoebox cages with soft bedding and enrichment and were maintained on a 12-h light/dark cycle in a humidity-controlled (40–60%) and temperature controlled (23°C) environment. Animals were provided unrestricted access to water and, until initiation of experimental diets, commercial diet (product code 5001; LabDiet). All animal protocols complied with NIH Care and Use of Laboratory Animal standards and were approved by the Rutgers University Institutional Animal Care and Use Committee (PROTO201702605).

### Study design and experimental diets

Mice were randomized to receive *ad libitum* access to one of four semi-purified, pelleted experimental diets (*n* = 20 for each diet) (for product details see: (Jonsson et al., 2021). In brief, mice were fed either Regular Fat (8% by weight, 18% of kcal) or High Fat (35% by weight, 60% of kcal) diets that were either SAA-sufficient Control or SAAR. High Fat SAAR diet contained slightly lower levels of methionine compared to the Regular Fat SAAR diet (0.12 vs. 0.17%). Diets were isocaloric and isonitrogenous compared to the respective control diet.

At the beginning of the experiment, mice were intraperitoneally injected with a bolus of 99% deuterium oxide (^2^H_2_O) in 0.9% saline relative to 60% of body weight. Mice had unlimited access to experimental diets and 8% ^2^H_2_O enriched drinking water for 1, 3, 7, 14, 21, or 35 days (*n* = 3/timepoint/diet, except for day 7 which had *n* = 5 animals/diet). This study design permits simultaneous monitoring of protein and DNA synthesis, and calculation of synthesis rate parameters (k) in response to acute and longer-term dietary interventions. Food was removed from all mice 4 hours prior to decapitation euthanasia. Brain, gastrocnemius, bone marrow, and the plasma fraction of blood were rapidly harvested, frozen in liquid nitrogen, and stored at -80°C until further analysis.

### Measurement of protein synthesis rates

Pre-frontal cortex was separated from whole brain. A cross section of gastrocnemius was obtained through the muscle mid-belly. Samples were pulverized under liquid nitrogen and 40–60 mg were aliquoted for assessment of alanine deuterium enrichment. Differential centrifugation was used to isolate cellular fractions enriched in cytosolic, mitochondrial, and mixed proteins; plasma samples were prepared as previously detailed (Miller et al., 2013; Drake et al., 2014). Protein fractions and plasma samples were cation exchanged and derivatized, dried, and resuspended in ethyl acetate for detection of deuterium enrichment via gas chromatography-mass spectrometry (GC-MS, Agilent Technologies, GC 5975C/MS7890A) using a DB5MS gas chromatograph column, helium as the carrier gas and methane as the reagent gas. The mass-to-charge ratios of 448, 449 (single-labeled), and 450 (double-labeled) representing pentafluorobenzyl-N,N-di (pentafluorobenzyl)alaninate derivatives were recorded, and quantified using MassHunter software (Agilent Technologies). The newly synthesized fraction of proteins was calculated at each timepoint using the enrichment of protein hydrolysates divided by the precursor alanine enrichment adjusted by the mass isotopomer distribution analysis as described previously (Hellerstein and Neese, 1999; Miller et al., 2013; Drake et al., 2014). From the fraction new, the rate of synthesis (k, 1/day) was calculated by curve fitting using a one-phase association (Reid et al., 2020).

### Measurement of DNA synthesis rates

Determination of deuterium incorporation into purine deoxyribose of DNA was achieved as described (Robinson et al., 2011). Briefly, DNA was isolated from bone marrow and from 15 mg samples of pulverized brain tissue using DNA mini extraction kits (QiAmp DNA, Qiagen 51306). DNA was incubated for 24 h with 50 μL of nuclease S1 (0.0025U/µl) and potato acid phosphatase (0.005U/µl) for 24 h. Hydrolysates were reacted with pentafluorobenzyl hydroxylamine and acetic acid and then acetylated with acetic anhydride and 1-methylimidazole. Dichloromethane extracts were dried, resuspended in ethyl acetate, and analyzed by GC-MS (Agilent Technologies, GC5975C/MS7890A; DB17MS column) as described (Busch et al., 2008). The fractional molar isotope at m/z 435 and 436 of deoxyribose was measured and quantified using MassHunter. The fraction new at each timepoint was calculated by comparison with bone marrow (representing an essentially fully turned-over cell population and thus the precursor enrichment) in the same animal (Drake et al., 2014). Day 1 was excluded from DNA synthesis analyses due to low deoxyribose deuterium enrichment in all diet groups. From the fraction new, the rate of synthesis (k, 1/day) was calculated using one-phase association curve fitting.

### Immunoblotting

Timepoints chosen for immunoblotting were days 1, 3, 7, and 35 to identify responses to both acute and long-term SAAR. 40–80 mg of brain and 15–20 mg of pulverized mixed gastrocnemius were suspended in 500 ml of RIPA buffer (150 mM sodium chloride, Triton X-100, 0.5% sodium deoxycholate, 0.1% SDS (sodium dodecyl sulfate), 50 mM Tris, pH 8.0 with phosphatase/protease inhibitors (HALT, Thermo Fisher). Brain samples were sonicated and muscle was homogenized using a Bullet Blender®. Protein concentration was determined via BCA assay. 30–45 μg of protein was loaded to a Tris-HCl gel for electrophoresis and transferred to PVDF. Membranes were blocked in 5% BSA in TBST prior to immunoblotting with the following primary antibodies diluted in 5% BSA in TBST at 4°C overnight: phospho-AMPKα [Thr172] 1:500 dilution (Cell Signaling Technology #2531, RRID:AB_330330), AMPKα 1:1000 dilution (Cell Signaling Technology #2532, RRID:AB_330331), RpS6 phospho-Ser [240/244] 1:500 dilution (Cell Signaling Technology #4858, RRID:AB_916156), Phospho-eIF2α (Ser51) (119A11) 1:1000 dilution (Cell Signaling Technology #3597, RRID:AB_390740), eIF2α (D7D3) 1:1000 dilution (Cell Signaling Technology #9079, RRID:AB_11178937) and RpS6 1:1000 dilution (Cell Signaling Technology #2217, RRID:AB_331355). Following TBST rinses, goat anti-rabbit horseradish peroxidase secondary antibody (Santa Cruz Biotechnology #sc-2004, RRID:AB_631746) diluted 1:10,000 in 5% BSA in TBST was applied. Proteins were visualized using enhanced chemiluminescent reagent and imaged. Immunoblotting for phosphorylated proteins was carried out first. Membranes were stripped and re-probed with antibodies for total proteins. Densitometry was performed for each diet and timepoint (*n* = 3) using AlphaView SA (ProteinSimple). Phosphorylated and total protein were normalized using ponceau staining and represented as the phospho-/total ratio.

### Statistics

Statistical analyses were performed using PRISM GraphPad 9. A two-way ANOVA with Fisher’s LSD post hoc test was used to assess the main effects of dietary fat and SAAR on protein and DNA synthesis. Rates of protein and DNA synthesis expressed as the kinetic parameter (k), were calculated via nonlinear regression of fraction new protein in brain and muscle using the mean value for the *n* = 3-5 animals at each timepoint for each dietary group. From the nonlinear regression, the slope of the curve (k) was calculated. Statistical significance was set at *p* < 0.05. While this study was powered to detect differences in protein synthesis rates, the primary outcome, it was underpowered for detecting statistical differences in protein content due to the small sample sizes (*n* = 3–5) at each timepoint for each dietary group. Therefore, protein content for each SAAR diet at selected timepoints was compared to control diet using unpaired t-tests and *p*-values between 0.05–0.10 were reported as trends.

## Results

### Brain protein synthesis rates are maintained in male mice fed a SAAR diet

**FIGURE 1:**
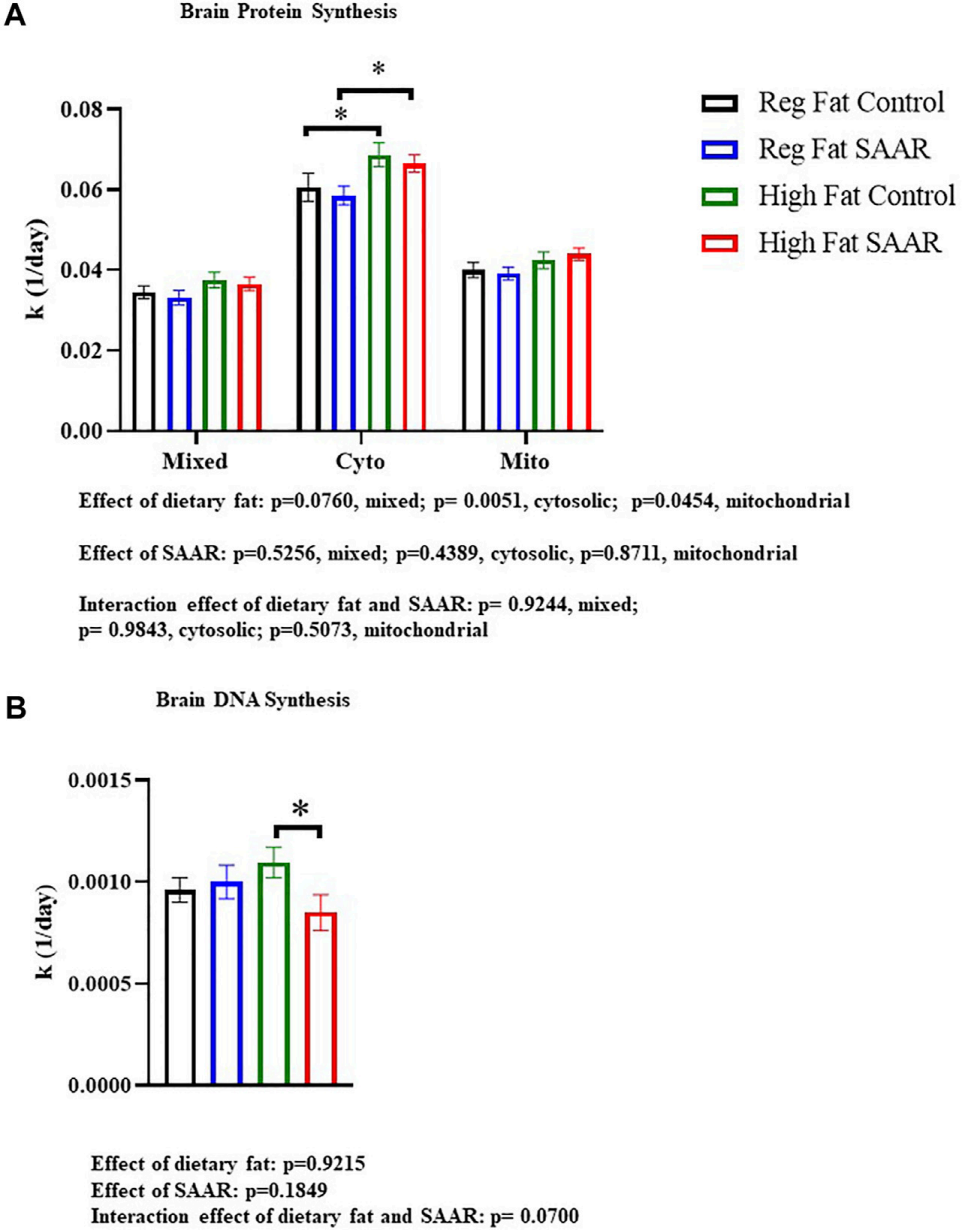
Rates of brain protein synthesis (K) in subcellular fractions and DNA synthesis in C57Bl/6J mice that were fed Regular Fat Control, Regular Fat SAAR, High Fat Control, or High Fat SAAR diets. Before starting their diets, mice were injected with a bolus of 99% ^2^H_2_O and had *ad libitum* access to drinking water enriched with 8% ^2^H_2_O. On days 1, 3, 7, 14, 21, and 35, *n* = 3-5 mice/timepoint were fasted for 4 hours and then sacrificed. A 1-phase association was used to calculate the rate of the rise of fraction new protein and DNA over time to determine the rate of synthesis (k, 1/d). **(A)** In the cytosolic and mitochondrial protein fractions, there was a main effect of dietary fat. Post hoc multiple comparisons showed that rates of protein synthesis were not significantly different in the mixed, cytosolic fractions, and mitochondrial fractions of mice fed Regular Fat SAAR and High Fat SAAR diets compared to their control diets. However, rates of cytosolic protein synthesis were greater in the High Fat Control compared to the Regular Fat Control, likely explaining the significant difference between High Fat SAAR and Regular Fat SAAR. **(B)** Post hoc analyses showed that mice fed a High Fat SAAR diet had significantly slower rates of DNA synthesis compared to High Fat Control. Protein and DNA synthesis data are presented as means ± SEM (*n* = 20 per diet group). Reg, regular; Cyto, cytosolic; Mito, mitochondrial, DNA, deoxyribonucleic acid. *p < 0.05.

Rates of brain protein synthesis were measured in mixed, cytosolic, and mitochondrial fractions of mice fed Regular Fat Control, Regular Fat SAAR, High Fat Control, and High Fat SAAR diets (Figure 1A). In the cytosolic and mitochondrial fractions, there was a main effect of dietary fat (*p* = 0.0051 cytosolic; *p* = 0.0454, mitochondrial). In the mixed and cytosolic fractions, there were no differences in rates of protein synthesis between Regular Fat SAAR (*p* = 0.6058 mixed fraction; *p* = 0.5934 cytosolic fraction) or High Fat SAAR (*p* = 0.7023 mixed fraction; *p* = 0.5743 cytosolic fraction), compared to their respective Control diets. Mitochondrial protein synthesis rates were maintained in High Fat SAAR compared to High Fat Control (*p* = 0.5595), as well as in Regular Fat SAAR compared to Regular Fat Control (*p* = 0.7228). Additionally, High Fat Control had greater rates of protein synthesis than Regular Fat Control (*p* = 0.0434) in the cytosolic fraction. Rates of DNA synthesis, reflecting cellular proliferation, were assessed simultaneously with protein synthesis to provide insight into allocation of newly synthesized proteins for newly proliferated cells versus for proteome maintenance (Figure 1B). High Fat SAAR group had significantly slower rates of DNA synthesis compared to High Fat Control (*p* = 0.0279).

### SAAR lowers rates of muscle protein synthesis in the gastrocnemius of male mice

**FIGURE 2:**
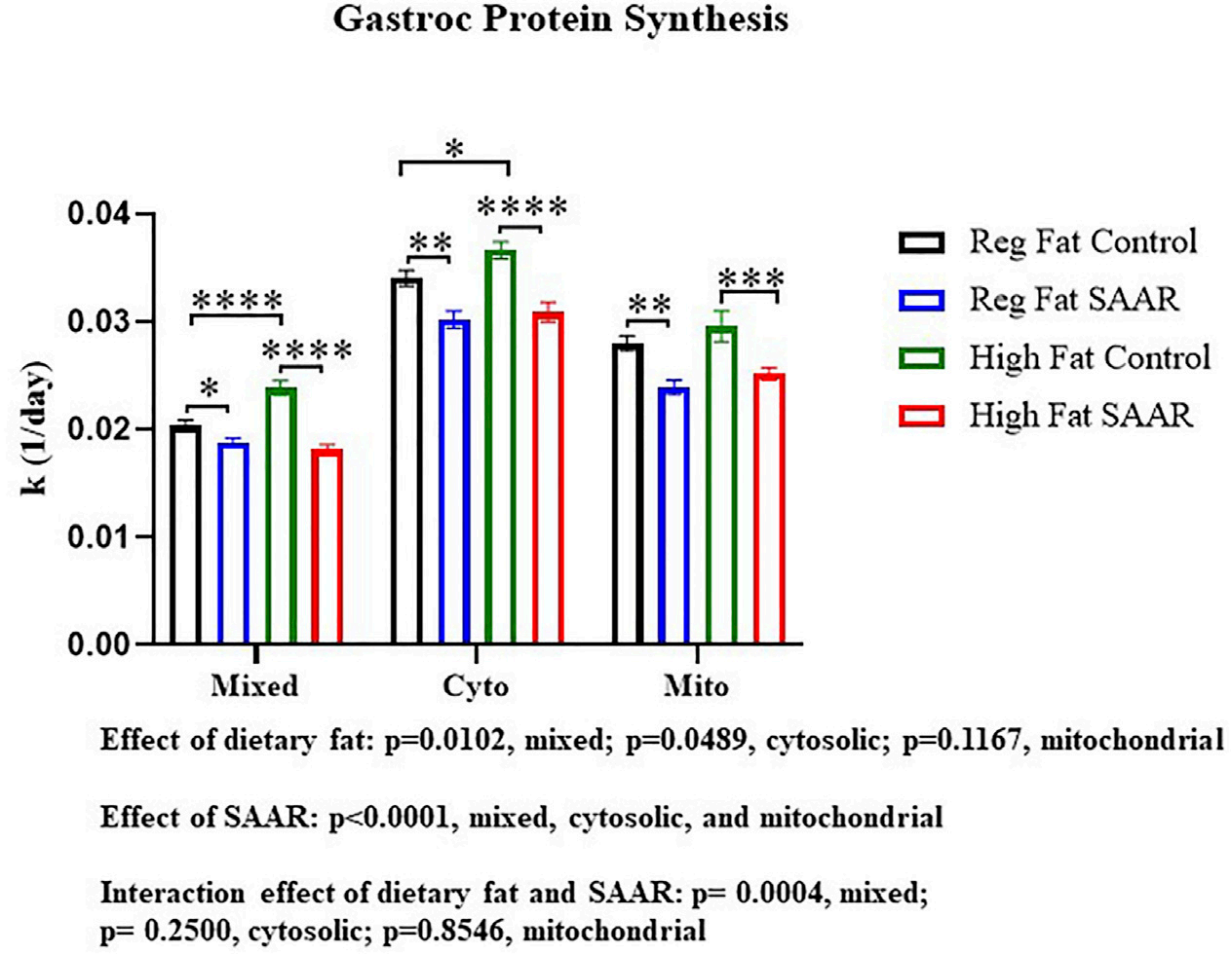
Rates of protein synthesis in gastrocnemius were measured in mice fed Regular Fat Control, Regular Fat SAAR, High Fat Control, or High Fat SAAR diets for 1, 3, 7, 14, 21, and 35 days. A 1-phase association was used to calculate the rate of the rise of fraction new protein synthesized over time, to determine the rate of synthesis (k, 1/d). There was a significant main effect of SAAR in the mixed, cytosolic, and mitochondrial protein fractions. Additionally, there was a significant main effect of dietary fat in the mixed and cytosolic fractions. The Interaction was significant in the mixed protein fraction only. Post hoc multiple comparisons showed that mice fed either of the SAAR diets had significantly slower rates of mixed, cytosolic, and mitochondrial proteins compared to the respective control diets. Additionally, rates of mixed and cytosolic protein synthesis were significantly different between the Regular Fat Control and High Fat Control diets. Data are presented as means ± SEM (*n* = 14–20); mixed and cytosolic fractions excluded day 1 and 3 (*n* = 3/day) due to low alanine deuterium enrichment. *p < 0.05, **p < 0.01, ***p < 0.001, ****p < 0.0001. SAAR diets are compared to their respective SAA sufficient control diets.

To contrast maintenance of protein synthesis in the brain, we assessed rates of protein synthesis in an energetically active peripheral tissue, gastrocnemius (Figure 2). There was a main effect of SAAR in all fractions (*p* < 0.0001) and a main effect of dietary fat in the mixed and cytosolic fractions (*p* < 0.05). The interaction between dietary fat and SAAR was significant in the mixed fraction (*p* = 0.0004). Both the Regular Fat SAAR (*p* = 0.0374) and High Fat SAAR (*p* < 0.0001) groups had slower rates of protein synthesis in the mixed fraction, comprised predominantly of myofibrillar proteins, compared to respective control groups. In the cytosolic fraction, rates of protein synthesis were significantly lower in both the Regular Fat SAAR (*p* = 0.0015) and the High Fat SAAR (*p* < 0.0001) groups compared to their controls. Mitochondrial rates of protein synthesis were also lower in the Regular Fat SAAR (*p* = 0.0019) and High Fat SAAR (*p* = 0.0009) groups compared to their controls. Additionally, High Fat Control had greater rates of protein synthesis than Regular Fat Control in the mixed (*p* < 0.0001) and cytosolic (*p* = 0.0288) fractions.

### Nutrient stress signaling in the brains of male mice varies in response to SAAR

**FIGURE 3:**
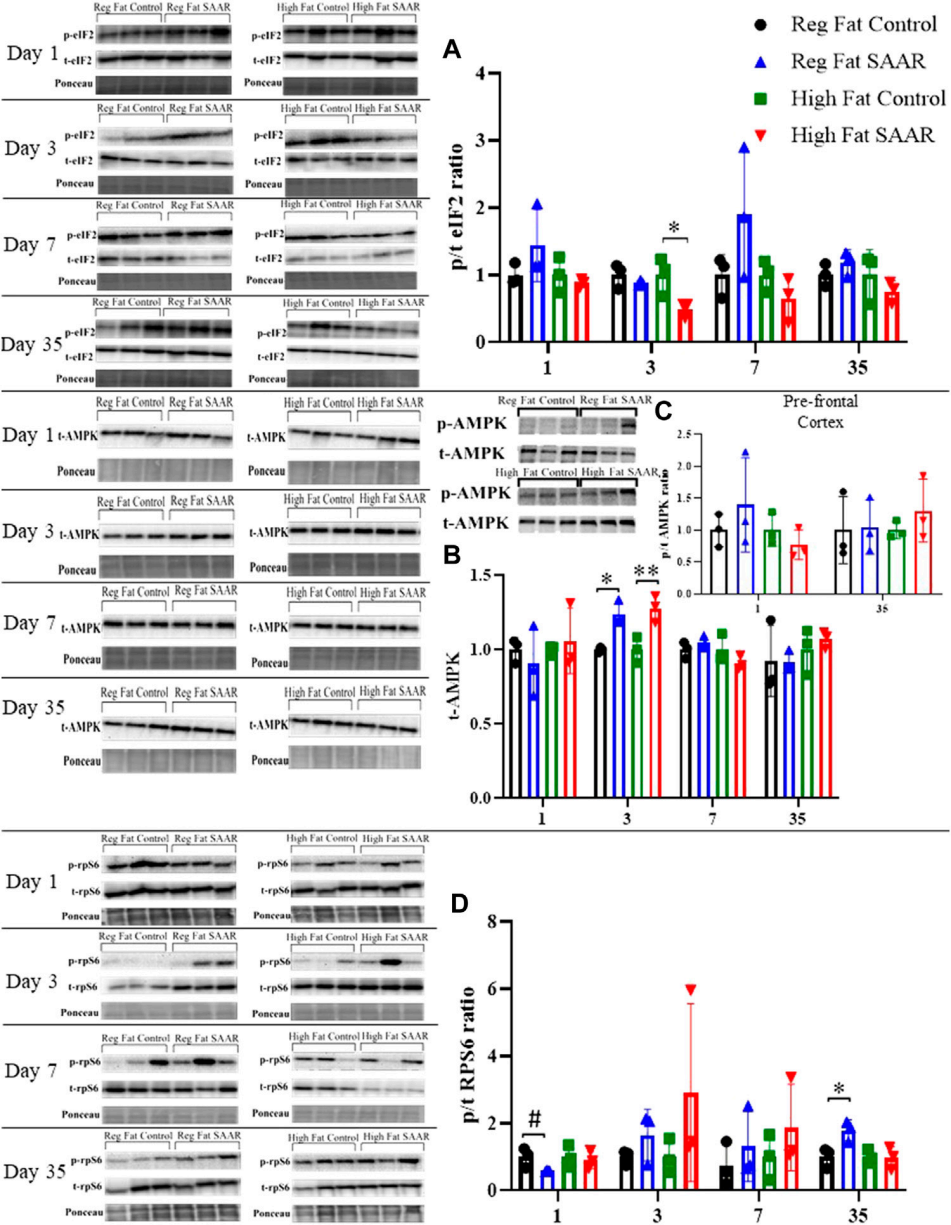
Immunoblot data for eIF2, AMPK, and rpS6 at days 1, 3, 7, and 35 of whole-brain homogenates. Phosphorylated (p) and total (t) proteins were detected and expressed as p/t ratios to reflect activation of proteins. Ponceau staining was used to normalize for total protein loading. **(A)** At day 3, mice fed a High Fat SAAR diet had significantly lower eIF2 activation compared to control. **(B)** Phosphorylated AMPK was undetectable in whole-brain homogenate; only t-AMPK is analyzed. At day 3, both the Regular Fat SAAR and High Fat SAAR groups had greater t-AMPK content than control. **(C)** Phosphorylated AMPK was detectable in pre-frontal cortex. Due to limited pre-frontal cortex tissue, only p/t ratios for day 1 and day 35 were assessed and no differences were observed. **(D)** rpS6 activation tended to be lower in the Regular Fat SAAR group at day 1, however, at day 35 was significantly greater than control. Data are presented as mean ± SD (*n* = 3/group). eIF2, eukaryotic initiation factor 2; AMPK; AMP-activated protein kinase; rpS6, ribosomal protein S6. *p < 0.05, **p < 0.01, # 0.05 < *p* < 0.10. SAAR diets are compared to their respective SAA sufficient control diets.

To complement our protein synthesis analyses, activation of stress signaling pathways including phosphorylated and total (expressed as a ratio) eIF2, AMPK, and rpS6 were assessed by immunoblotting. Due to insufficient amounts of pre-frontal cortex tissue, the remainder of the brain was homogenized. Days 1, 3, and 7 were chosen to measure acute responses to SAAR, while day 35 was used to measure long-term responses. Activation of eIF2 (Figure 3A) was significantly lower at day 3 in the High Fat SAAR group (*p* = 0.0460) compared to High Fat Control. Phosphorylated AMPK was not observed in whole-brain homogenates, but total AMPK (Figure 3B) was significantly greater at day 3 in both Regular Fat SAAR (*p* = 0.0080) and High Fat SAAR (*p* = 0.0180) compared to their respective Control diets. Phosphorylated AMPK was detected in pre-frontal cortex (Figure 3C) at day 1 and day 35. Phosphorylation of rpS6 (Figure 3D) trended lower in Regular Fat SAAR (*p* = 0.0690) as compared to Regular Fat Control. But at day 35, rpS6 phosphorylation was significantly greater in Regular Fat SAAR (*p* = 0.0339).

### Major nutrient stress signaling is minimally altered by SAAR in gastrocnemius

**FIGURE 4:**
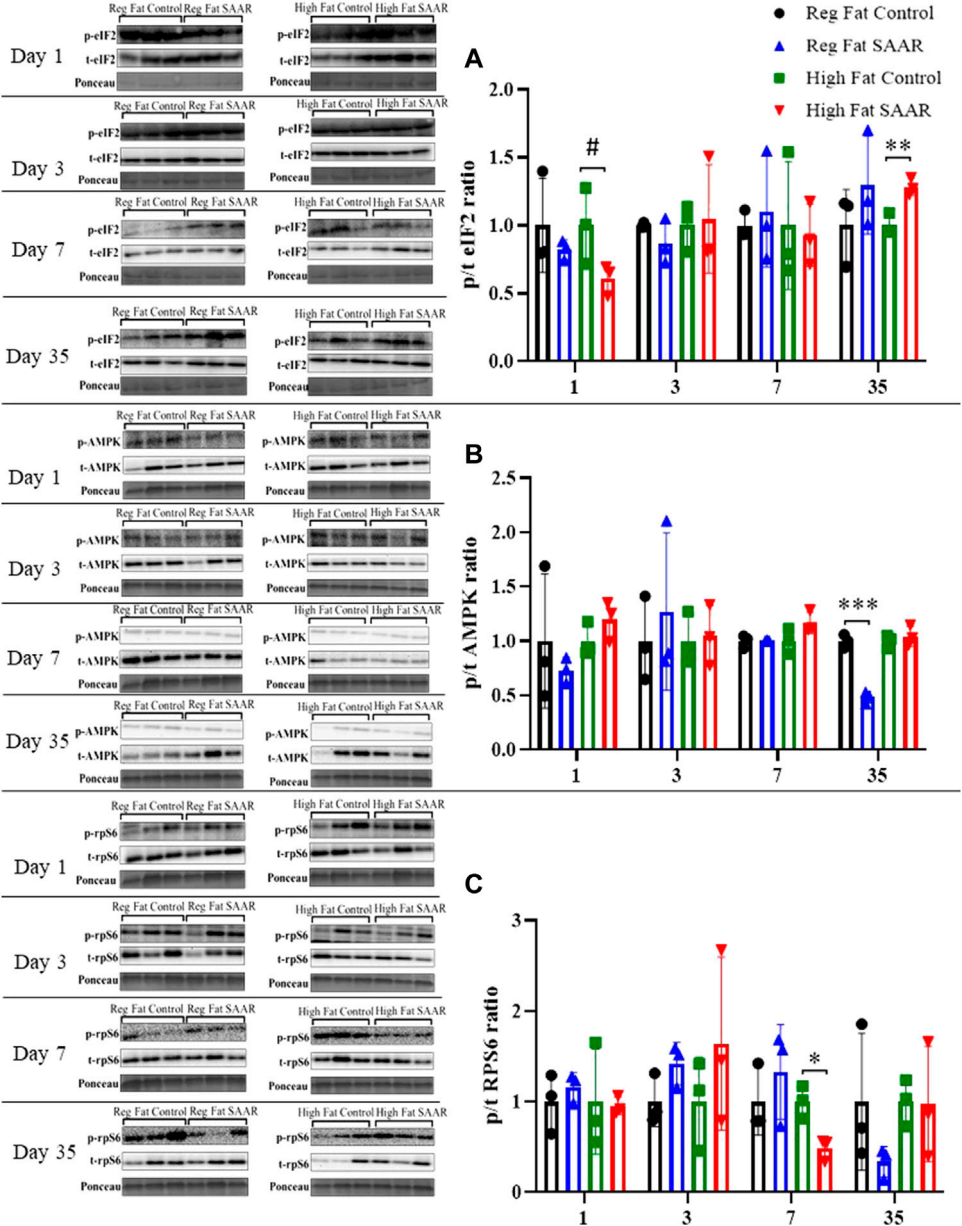
Immunoblot data for eIF2, AMPK, and rpS6 at days 1, 3, 7, and 35 in gastrocnemius homogenates. **(A)** At day 1, mice fed a High Fat SAAR diet tended to have lower eIF2 activation than control. By day 35, mice fed a High Fat SAAR diet had greater eIF2 activation. **(B)** AMPK activation was significantly lower in mice fed a Regular Fat SAAR diet at day 35. There were no significant differences at other timepoints for other groups. **(C)** rpS6 activation was significantly lower in mice fed a High Fat SAAR diet at day 7 compared to control, however, no significant differences were detected at other timepoints and diets. Data are presented as mean ± SD (*n* = 3/group). eIF2, eukaryotic initiation factor 2; AMPK; AMP-activated protein kinase; rpS6, ribosomal protein S6. *p < 0.05, **p < 0.01, ***p < 0.001, # 0.05 < *p* < 0.10. SAAR diets are compared to their respective methionine sufficient control diets.

Phosphorylated and total eIF2, AMPK, and rpS6 content was assessed via immunoblotting for each diet and at both acute and chronic (Days 1, 3, 7, and 35) timepoints in gastrocnemius (Figure 4). eIF2 phosphorylation (Figure 4A) trended lower at day 1 (*p* = 0.0878) in the High Fat SAAR group as compared to High Fat Control. No significant differences were observed between SAAR and Control diets at days 3 and 7. At day 35, the High Fat SAAR group had significantly greater eIF2 phosphorylation as compared to its Control (*p* = 0.0082). AMPK activation (Figure 4B) was significantly lower in the Regular Fat SAAR group as compared to Control (*p* = 0.0004) at day 35, whereas rpS6 phosphorylation (Figure 4C) was significantly lower (*p* = 0.0149) in the High Fat SAAR as compared to Control at day 7.

## Discussion

### Summary of findings

In this brief report, we examined the impact of dietary SAAR on rates of protein synthesis, DNA synthesis, and activation of nutrient stress signalling proteins in two energetically demanding tissues, the brain and gastrocnemius. We found that both SAAR diets decreased rates of protein synthesis in all fractions of gastrocnemius. In contrast, rates of protein synthesis in all fractions of pre-frontal cortex were maintained in mice fed either a Regular Fat or High Fat SAAR diet compared to SAA-sufficient controls. Rates of DNA synthesis were assessed simultaneously with protein synthesis in brain pre-frontal cortex. Mice fed High Fat SAAR showed slower rates of cellular proliferation, indicating that there was a greater allocation of newly synthesized proteins for proteome maintenance rather than for newly proliferating cells.

### Brain is resistant to perturbations in protein synthesis under SAAR

Maintenance of proteostasis is essential for cell function and loss of proteostasis is a key driver of aging in both brain and skeletal muscle (Balch et al., 2008; Lopez-Otin et al., 2013; Sweeney et al., 2017; Mattson and Arumugam, 2018; Fernando et al., 2019). Proteome maintenance requires cooperation of a complex network of mechanisms (Verma et al., 2021). In addition to the requirement of protein synthesis for new cell proliferation, the limited cellular capacity for protein repair makes protein turnover a critical part of proteostatic maintenance especially with advancing age (Chondrogianni et al., 2014). In our previous work with experimental models of healthspan extension including SAAR, rapamycin treatment, growth hormone disruption, and caloric restriction, we have shown a trade-off of protein synthetic resources towards somatic maintenance over cellular proliferation (Drake et al., 2013; Miller et al., 2013; Drake et al., 2015; Pettit et al., 2017; Jonsson et al., 2021). Additionally, a shared trait of our published experimental models of healthspan extension is a maintenance of the mitochondrial proteome despite suppression of global protein synthesis. For example, we reported that in mice fed the SAAR diets used in the current study, rates of hepatic mitochondrial protein synthesis are maintained despite slower rates protein synthesis in cytosolic and mixed protein fractions compared to controls (Jonsson et al., 2021).

Because loss of proteostasis is a pathology contributing to brain aging and age-related neurodegeneration, we posited that experimental SAAR may have similar effects in brain as we observed in liver (Pettit et al., 2017; Sweeney et al., 2017; Mattson and Arumugam, 2018; Jonsson et al., 2021; Verma et al., 2021). Despite its relatively small mass, the brain accounts for ∼20% of whole-body energy expenditure at rest (Camandola and Mattson, 2017). Interventions that promote proteostasis in the brain, particularly mitochondrial proteostasis, have the potential to promote successful brain aging (Hetz, 2021). Accordingly, SAAR delays age-related cognitive decline and preserves synaptic integrity through an increase in brain mitochondrial biogenesis (Ren et al., 2021). Therefore, it was not surprising to observe that rates of brain mitochondrial protein synthesis, a true measurement of mitochondrial biogenesis, were maintained under both SAAR diets in our study. However, in contrast to our past reports that SAAR slows rates of protein synthesis in cytosolic and mixed fractions of hepatic tissue, both of those fractions were also maintained in brain during SAAR. Based on this finding, we can conclude that in response to SAAR, the brain is resistant to a reduction in protein synthesis, likely to maintain essential neuronal functions (Rangaraju et al., 2019). This finding also underscores tissue specificity in the proteostatic response to nutrient stress. An important consideration when assessing rates of protein translation as a proteostatic mechanism, is allocation of newly synthesized proteins for cell proliferation (Hamilton and Miller, 2017). Here, in addition to showing that rates of protein synthesis were maintained in all fractions of the pre-frontal cortex, cellular proliferation was significantly lower in the High Fat SAAR group, suggesting greater allocation of newly synthesized proteins towards proteome maintenance rather than growth, a finding that is consistent with other healthspan-extending treatments we have studied (Drake et al., 2013; Drake et al., 2014; Drake et al., 2015).

Our finding that pre-frontal cortex rates of protein synthesis are maintained in all fractions under SAAR, prompted us to compare this outcome to another energetically active peripheral tissue; gastrocnemius muscle. While several factors influence the contribution of skeletal muscle mass to total energy expenditure at rest, a generally accepted estimate is ∼20%; muscle contraction, of course, increases the contribution of this tissue to total energy expenditure (Heymsfield et al., 2021). We previously reported that during 21 days of SAAR, during which time total energy expenditure was increased compared to controls, rates of cytosolic and mixed protein synthesis in gastrocnemius were reduced, similar to our current findings (Pettit et al., 2017). However, we found that the rates of muscle mitochondrial protein synthesis were maintained, again suggesting that there may be selective translation of mitochondrial proteins, particularly in response to energy stresses (Pettit et al., 2017). In contrast, both levels of SAAR in the current study resulted in slower rates of protein synthesis in all fractions of gastrocnemius. A few differences between our previous and current studies could potentially contribute to differences observed in the two studies. First, the longest duration of the intervention (21 days in the previous study versus 35 days in the current report) varied between the experiments. Second, in the previous study mice were fasted overnight before harvesting tissues whereas in this study food was removed for only 4 h before tissue collection. Additionally, in the current study, we pulverized the gastrocnemius and plantaris together whereas in the previous study only the gastrocnemius was assessed. Finally, a high dietary fat content increased rates of protein synthesis compared to a regular fat content (60% fat vs. 18% fat) in the cytosolic fraction of brain and in the mixed/cytosolic fractions of gastrocnemius, with evidence suggesting interaction between the effects of dietary fat and SAAR in the muscle. This observation is consistent with our previous study in which high-fat feeding increased protein synthesis in the mitochondrial and cytoplasmic fractions of quadriceps (Newsom et al., 2017). However, the mechanisms explaining the interaction of high-fat feeding and SAAR on proteostasis requires further exploration.

### Classic nutrient/energy sensing proteins are minimally activated in brain and muscle in response to SAAR

Restriction of essential amino acids has been repeatedly reported to phosphorylate eIF2 leading to initiation of the integrated stress response via the GCN2 kinase leading to suppression of protein synthesis (Zhang et al., 2002; Anthony et al., 2004). Similarly, insufficiency of essential amino acids can activate the cellular energy sensor AMPK, signaling for translation suppression (Hardie, 2011). We previously reported that despite reducing rates of hepatic protein synthesis, feeding a SAAR diet did not increase phosphorylated-eIF2 in liver and, further, deletion of GCN2 did not alter suppression of hepatic protein synthesis in response to SAAR (Jonsson et al., 2021). Here, we found inconsistent evidence of activation of nutrient sensing pathways. In brain, eIF2 activation was lower in the High Fat SAAR group and total AMPK content was greater in both SAAR groups compared to controls at day 3. Activation of rpS6 was greater in Regular Fat SAAR at day 35. In gastrocnemius, we found modest differences, with a decrease in eIF2 activation day 1 and increased activation after 35 days of High Fat SAAR. AMPK activation was lower in Regular Fat SAAR compared to control at day 35. Finally, rpS6 activation was only lower in the High Fat SAAR compared to control at day 7. As mentioned, the experimental design employed in our study was specifically chosen to identify changes in kinetic protein synthetic responses to SAAR treatment and, as such, was not powered to detected differences in content of signaling proteins at individual timepoints. This should be taken into consideration when forming conclusions about tissue nutrient sensing in response to SAAR. However, our findings that nutrient stress signaling is minimally altered in the brain with SAAR are consistent with the observation that rates of brain protein synthesis are not altered with SAAR.

### Conclusions and next steps

This brief report provides the first observation that, unlike previous assessments in liver, rates of protein synthesis in all fractions of the pre-frontal cortex are maintained, with little evidence of activated nutrient stress signaling, during SAAR. This is in contrast to a peripheral tissue, gastrocnemius, where we observed suppression of protein synthesis with both levels of SAAR. Next steps include identifying the influence of both sex differences and age on responses to SAAR in the brain and, potentially, animals modified to be susceptible to progressive neurodegeneration. Our priorities include coupling assessments of protein turnover with protein misfolding, mitochondrial function, cognitive function, and other potentially critical cell signalling changes during SAAR.

## Data Availability

The raw data supporting the conclusions of this article will be made available by the authors, without undue reservation.

## Abbreviations

SAAR: sulfur amino acid restriction; GCN2, general control nonderepressible factor 2; EIF2, eukaryotic initiation factor 2; ATF4, activating transcription factor 4; FGF21, fibroblast growth factor 21; AMPK, AMP-activated kinase; mTOR, mechanistic target of rapamycin; rpS6, ribosomal protein S6.
